# Clinical, Serological, and Genetic Characteristics of a Hungarian Myositis-Scleroderma Overlap Cohort

**DOI:** 10.1155/2022/6251232

**Published:** 2022-05-02

**Authors:** Katalin Szabó, Levente Bodoki, Melinda Nagy-Vincze, Tibor Béldi, Anett Vincze, Erika Zilahi, József Varga, Gabriella Szűcs, Katalin Dankó, Zoltán Griger

**Affiliations:** ^1^Division of Clinical Immunology, Faculty of Medicine, University of Debrecen, Móricz Zs. krt. 22, 4032 Debrecen, Hungary; ^2^Division of Rheumatology, Faculty of Medicine, University of Debrecen, Móricz Zs. krt. 22, 4032 Debrecen, Hungary; ^3^Department of Laboratory Medicine, Faculty of Medicine, University of Debrecen, Nagyerdei krt. 98, 4032 Debrecen, Hungary; ^4^Department of Medical Imaging, Division of Nuclear Medicine, University of Debrecen, Debrecen, Hungary

## Abstract

Overlap myositis is a distinct subgroup of idiopathic inflammatory myositis (IIM) with various clinical phenotypes. The aim of this study was to determine the clinical, serological, and genetic features of systemic sclerosis (SSc)-IIM overlap patients. It was a retrospective study using clinical database of 39 patients, fulfilling both the criteria of SSc and IIM. 56.4% of the patients had limited cutaneous, 43.6% had diffuse cutaneous SSc, whereas 7.7% of the patients had dermatomyositis and 92.3% polymyositis. The two diseases occurred simultaneously in 58.97%, while 10.26% in myositis and 30.77% in scleroderma were initially diagnosed. The frequencies of organ involvement were interstitial lung disease 71.8%, dysphagia 66.7%, cardiac involvement 41%, pulmonary arterial hypertension (PAH) 30.8%, and renal involvement 12.8%, respectively. The presence of human leukocyte antigen (HLA) − DRB1∗03 and DQA1∗051∗01 alleles were significantly higher in the overlap patients than in healthy controls (82.35% vs. 27.54%; *p* < 0.0001 and 88.24% vs. 30.16; *p* < 0.0001). Certain clinical parameters, such as fever at diagnosis (41.67% vs. 7.41%, *p* = 0.0046), cardiac involvement (83.33% vs. 22.22%, *p* = 0.0008), subcutaneous calcinosis (41.66 vs. 11.11, *p* = 0.01146), and claw hand deformity (25% vs. 11.11%, *p* = 0.00016) were significantly associated with the presence of PAH. Upon comparison, the overlap patients and anti-Jo-1 positive antisynthetase patients showed similarities in terms of genetic results and major clinical features; however, SSc-IIM overlap patients could be distinguished with higher erythrocyte sedimentation rate (ESR) level, more frequent presence of Raynaud's phenomenon (*p* < 0.0001; OR: 20.00), dysphagia (*p* < 0.0001; OR: 15.63), and infrequent livedo reticularis (*p* < 0.01; OR: 0.11). SSc-IIM overlap myositis is a unique group within IIM-s possessing characteristic clinical features.

## 1. Introduction

Idiopathic inflammatory myopathies (IIMs) consist of rare heterogeneous autoimmune disorders that present with marked proximal and symmetric muscle weakness accompanied by the involvement of various internal organs such as the lungs, heart, and esophagus [1]. Traditionally, IIMs are classified into three major subtypes, polymyositis (PM), dermatomyositis (DM), and inclusion body myositis (IBM), but recently, other subgroups, such as necrotizing autoimmune myopathy (NAM) and antisynthetase syndrome (ASSD), have been also identified [2]. Inflammatory myositis can occur together with other connective tissue diseases (CTD) too, broadly known as overlap myositis (OM). Autoantibodies are hallmarks of IIMs and are strongly associated with clinical features and can be used to define phenotypic subtypes [3]. Thus, in the past two decades, most cohort-based studies have been on DM and PM, or the presence of a distinct autoantibody thereby limiting the understanding of OM. However, the prevalence of overlap myositis in inflammatory myopathies varies from 22 to 49% [4–6].

Overlap myositis is constituted by myositis occurring in the setting of systemic lupus erythematosus (SLE), systemic sclerosis (SSc), mixed connective tissue disorder (MCTD), rheumatoid arthritis (RA), and Sjögren's syndrome (SS). One of the most frequent overlap myositis is represented by myositis and SSc, comprising 15-42.6% of all the overlap syndrome IIMs [6–8]. On the contrary, muscle disease, or myopathy, in scleroderma has been thought to be a relative bystander in comparison to other organ disease manifestations. Due to a lack of classification criteria of muscle disease in scleroderma, the prevalence of muscle disease in scleroderma varies widely [9, 10]. There is no consensus on whether an inflammatory myopathy in SSc should be considered as disease symptom, or as scleroderma-myositis overlap, but it is becoming more apparent that SSc patients with concomitant muscle disease have poorer outcomes including disability and death [11, 12]. Hence, the clinic-serological identification and early recognition of an IIM-SSc overlap situation is useful to clarify prognosis and facilitate management.

It is well known that genetics could have a crucial role in the pathomechanism of the diseases and is associated with the presence of certain autoantibodies and clinical phenotype [13]. Patients with systemic sclerosis and myositis have characteristic genetic features, such as the presence of HLA DRB1∗1104, DQA1∗05 : 01, and DQB1∗03 : 01 in SSc and HLA DRB1∗03 : 01 and DQA1∗05 : 01 in myositis [14–16]. Furthermore, clinical characteristics of patients with ASSD and IIM-SSc overlap patients might be similar [6]. The presence of mechanic's hand, interstitial lung disease, Raynaud's phenomenon, and myositis is frequently found in both clinical syndromes. There is also recent literature data that supports the common pathogenic origin of PM/Scl OM and ASSD [14, 17]. However, the genetic features of IIM-SSc overlap patients are not known; thus, comparing clinical profile and genetic features of patients with ASSD and IIM-SSC OM might help us better understand the pathomechanism of this disease.

Therefore, the aims of this study were (1) to determine the demographic, clinical, serological, laboratory, and genetic features of Hungarian scleroderma-myositis overlap patients; (2) to define and compare the HLA haplotype of the overlap patients with anti-Jo-1-positive ASSD patients and healthy controls; and (3) to assess relevant markers or clinical features at the onset of the disease, which can predict the progression of myositis or occurrence of major organ involvement.

## 2. Materials and Methods

### 2.1. Patients

In our retrospective study, all SSc-myositis overlap patients were selected from the Hungarian patient database used at the University of Debrecen. A total of 414 patients were first selected, having at least one of the following International Classification of Diseases (ICD)-10 diagnosis codes: M34: systemic sclerosis; M34.8: other forms of systemic sclerosis; M34.9: systemic sclerosis, unspecified; M33: dermatopolymyositis; M33.1: other dermatomyositis; M33.2: polymyositis; M60.1: interstitial myositis; M60.8: other myositis; M60.9: myositis, unspecified. All identified patients were treated at the Department of Clinical Immunology or at the Department of Rheumatology and had at least two outpatient clinic appearances between January 2001 and January 2019. Secondly, these patients were sorted by those who fulfilled the diagnostic criteria for scleroderma (according to 1980 American Rheumatology Association (ARA) [18] or 2013 American College of Rheumatology/European League Against Rheumatism (ACR/EULAR) [19]) and had a definitive or probable diagnosis for IIM (muscle weakness, high muscle enzyme levels, plus positive electromyography (EMG) and/or positive muscle biopsy in polymyositis or skin symptoms in dermatomyositis, according to Bohan and Peter [20]/or probable/definitive IIM according to the EULAR/ACR myositis criteria [21]). Patients without characteristic dermatomyositis skin symptoms were only classified as having muscle involvement, if EMG/or muscle biopsy was performed. Based on these criteria, 39 cases of scleroderma-myositis overlap could be identified. Interstitial lung disease was defined as presented by radiographic findings (high-resolution computed tomography (HRCT)) and pulmonary function tests (spirometry, diffusing capacity for carbon monoxide). Dysphagia was diagnosed by barium radiography of the esophagus. Intestinal involvement except dysphagia was determined based on the presence of either malabsorption, chronic diarrhea/small intestinal bacterial overgrowth, or motility disturbance. Cardiac involvement, indicated by relaxation abnormalities, conduction disturbances, or right ventricular hypertrophy, was assessed by electrocardiogram (ECG), two-dimensional, and Doppler echocardiography. Cardiac involvement was encoded in case of pericarditis, myocarditis, conduction disturbances, myocardial ischemia on ECG, and recurrent arrhythmia. Pulmonary artery systolic pressure (PASP) was calculated with the measurement of the tricuspid regurgitation peak velocity in those patients that had some degree of tricuspid regurgitation. Mean values of measurements from 3 consecutive beats were calculated. The diagnosis of PAH was set up, when estimated PASP was ≥30 mmHg.

### 2.2. Immunoserology

Immunological analyses included tests for the following autoantibodies. Antinuclear antibodies (ANA), anticentromere antibody (ACA), antihistone antibodies, and anticytoplasmic antibodies were determined by indirect immunofluorescence on HEp-2 cells (Viro-Immun Labor-Diagnostika GmbH, Oberursel, Deutschland); ANA positivity was assessed at 1: 40 dilution. Anti-Scl70, anti-Sm, and anti-Sm/RNP were determined in all patients by enzyme-linked immunosorbent assay (ELISA) (Hycor Biomedical Inc., Garden Grove, CA, USA). Anti-Jo-1, anti-Mi-2, anti-Pm-Scl, and anti-Ku antibodies were detected by membrane-fixed immunoblot (ORGENTEC Diagnostika GmbH, Mainz, Deutschland). Anti-Sjögren's-syndrome-related antigen A (SSA) and anti-Sjögren's-syndrome-related antigen B (SSB) were determined by ELISA (Hycor Biomedical Inc., Garden Grove, CA, USA), as well as anti-U1RNP (ORGENTEC Diagnostika GmbH, Mainz, Deutschland). IgM rheumatoid factor (RF) was assessed by nephelometry (DIALAB GmbH, Neudorf, Austria). The normal value for IgM RF was <50 U/ml. ELISA was used for the measurement of the following autoantibodies: anticyclic citrullinated peptide (CCP) (Euro Diagnostica AB, Lundvagen, Sweden), anti-double-stranded deoxyribonucleic acid (dsDNA), anti-Beta-2-glycoprotein I (B2GPI), anticardiolipin, and antiphospholipid antibodies (ORGENTEC Diagnostica GmbH, Mainz, Deutschland). These commercially available methods were used following the manufacturer's protocol. Antiendothelial cell antibodies (AECA) were determined by a home-made ELISA at the Department of Laboratory Medicine, University of Debrecen.

### 2.3. Genotyping

Peripheral blood from SSc-myositis overlap patients and 69 healthy individuals was collected in ethylenediaminetetraacetic acid (EDTA) Vacutainers. High molecular weight deoxyribonucleic acid (DNA) was extracted from these blood samples for genotyping. With the manufacturer's recommendation, genomic DNA was extracted, using the QIAamp DNA Blood Mini Kit-QIAGEN GmbH, Germany. For DNA quantification, ultraviolet absorption was performed at 260 and 280 nm and stored at -20°C until analyzed. Human leukocyte antigen (HLA) class II alleles were genotyped at the HLA-DQA1 and HLA-DRB1 loci using sequence-specific primers (GenoVision, Olerup SSP, Oslo, Norway). All samples were processed on polymerase chain reactions (PCR) according to the manufacturer's instructions. HLA genotypes were specified on the basis of the PCR product pattern obtained using 2% agarose gel electrophoresis.

### 2.4. Statistical Analysis

Statistical analysis was made using the SPSS 27 statistical software. For contingency tables, Fisher's exact test or the chi-square (*χ*2) test was applied. The values of continuous variables in different groups were compared with either Welch's *d*-test or the nonparametric Mann–Whitney test, depending on the result of the normality test (Shapiro-Wilk). During statistical analysis, the *p* value less than 0.05 was regarded as statistically significant; for the pairwise comparison of more than two groups, Bonferroni's or Hochberg's correction was applied.

## 3. Results

### 3.1. Demographic Data, Organ Involvements

Data of 39 myositis-SSc overlap patients were evaluated.  Table 1 shows the demographic, clinical, and laboratory characteristics of the patients. The mean age at diagnosis was 42.0 ± 14 (13-76) years, and 56.41% of the overlap patients (*n* = 22) had limited cutaneous (lcSSc), 43.59% (*n* = 17) diffuse cutaneous systemic sclerosis (dcSSc), 92.31% (*n* = 36) PM, and 7.69% (*n* = 3) DM. The two diseases occurred simultaneously in 23 patients (58.97%), while 4 cases (10.26%) in myositis and 12 cases (30.77%) in scleroderma were initially diagnosed, before the onset of the other disease. When not occurring simultaneously, the first diagnose was followed by the second disease in 2.9 (1-6) years.

Raynaud's phenomenon (RP) was present in 38 patients (97.44%) at disease onset. The frequency of the most important internal organ manifestations during disease progression were the following: interstitial lung disease (ILD) 28 patients (71.79%), dysphagia 26 patients (66.67%), intestinal involvement (except dysphagia) 11 patients (28.21%), cardiac involvement 16 patients (41.03%), pulmonary arterial hypertension (PAH) 12 patients (30.77%), and renal involvement 5 patients (12.82%). Fever at diagnosis was present in 7 (17.95%) cases. Regarding skin symptoms, mechanic's hand could be detected in 5 cases (12.82%), subcutaneous calcinosis in 8 cases (20.51%), and livedo reticularis in two (5.13%) cases. The average creatine kinase (CK) at diagnosis was 1542.37 ± 1975.33 U/l, and the average lactate dehydrogenase (LDH) was 725.67 ± 360.58 U/l.

Comparison of these results with historical data of our 49 anti-Jo-1-positive patients [22] showed similar age at diagnosis, male/female ratio, average CK, or LDH levels at diagnosis, but ESR levels at diagnosis was significantly higher in overlap group (40.26 vs. 24.24 mm/h; *p* < 0.01). Twenty-eight binary variables (symptoms and laboratory parameters) were compared between the 2 groups with Fisher's exact test, applying Hochberg's correction for multiple comparisons. The frequency of the 3 parameters was significantly different (Table 1), the presence of Raynaud's phenomenon (*p* < 0.001, odds ratio (OR) 20.00; 95% confidence interval (CI): 2.54 to 166) and dysphagia (*p* < 0.0001, OR: 15.6; 95% CI: 5.21 to 45.5) was more, whereas livedo reticularis (*p* < 0.01, OR 0.11; 95% CI: 0.02 to 0.52) was less frequently detected in the overlap group than in the anti-Jo-1-positive patients.

**Figure 1:**
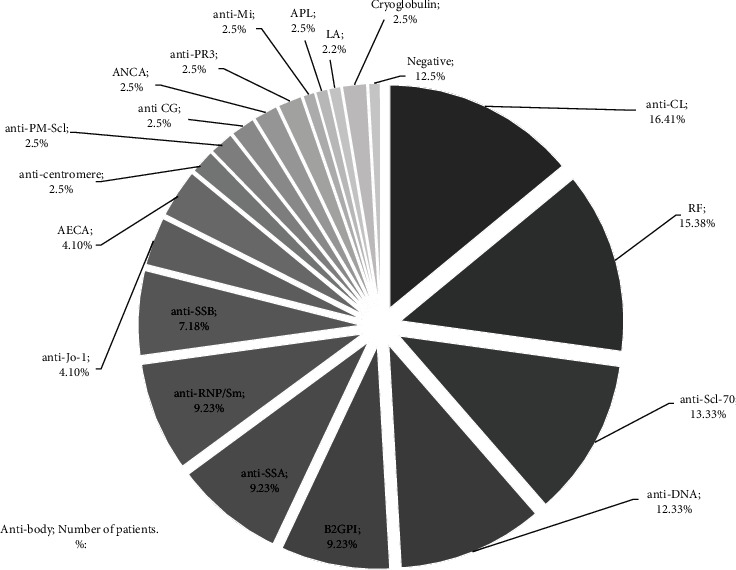
Autoantibody profile of the investigated population.

Examining the immune-serological parameters, we observed very heterogeneous results. All of our overlap patients were antinuclear antibodies (ANA) positive, and detailed antibody positivity can be followed on Figure 1. None of the autoantibodies could be identified as a marker for this overlap. There was no anti-Ku or anti-U1-RNP-positive patient.

### 3.2. Genetics

**Figure 2:**
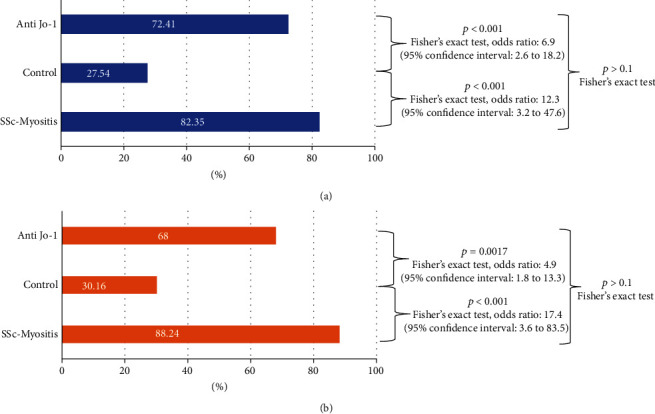
Genetic results of the investigated SSc-myositis overlap population: (a) the presence of HLA − DRB1∗03 genotype; (b) the presence of HLA − DQA1∗05 : 01 genotype. Significances of the incidence of both DQA1∗05 : 01 and DRB1∗03 genotypes in the 3 disease groups were calculated with chi-square test. For multiple (3) pairwise comparisons, the Bonferroni correction was applied, so that the level of significance was set to 0.017. Significances of the presence of genotypes between both two groups were calculated with Fisher's exact test.

HLA-DRB1 and DQA1 genotypes of 17 patients with myositis-SSc overlap and 69 healthy individuals were determined using commercial sequence-specific oligonucleotide kits. The most frequent HLA-DRB1 (HLA − DRB1∗03) alleles were present in 14 (82.35%) overlap patients and 19 (27.54%) in controls. Additionally, HLA − DQA1∗05 : 01 alleles were detected in 17 (88.24%) patients and 19 (30.16%) in controls (Figure 2). A comparison of these results showed that the presence of DRB1∗03 was significantly more frequent in the overlap than in the healthy control group (Fisher's exact test: *p* < 0.0001; OR: 12.3; 95% CI: 3.2 to 47.6). Similar results were seen with the frequency of DQA1∗05 : 01 (Fisher's exact test: *p* < 0.0001, OR: 17.4; 95% CI: 3.6 to 83.5). We then compared this data with genetic results of our anti-Jo-1 ASSD patients [22], but found no significant difference between the frequency of neither the DQA1∗05 : 01 nor the DRB1∗03 genotype in the overlap and anti-Jo-1 group (Fisher's exact test: *p* > 0.1; Figure 2). The presence of HLA − DRB1∗03 − HLA − DQA1∗05 : 01 haplotype in overlap patients with different parameters of the disease phenotype (organ involvement, laboratory parameters, and serological status) were also investigated; however, significant correlations could not be detected (data not shown).

### 3.3. Comparison of Patients with and without Pulmonary Arterial Hypertension (PAH)

PAH, which represents a significant factor in SSc disease progression [23, 24] was detected in 30.77% (12/39 patients) of our overlap population. Looking for predictive factors of PAH, we compared the clinical and laboratory findings of the PAH positive group with PAH-negative patients (Table 2). We found that the presence of ILD (75% vs. 77.78%), arthritis (83.33% vs. 81.48%), Raynaud phenomenon (100% vs.96.3%), and dysphagia (75% vs. 62.96%) was quite similar in the PAH-positive and PAH-negative group. In contrast, fever at diagnosis (41.67% vs. 7.41%, *p* = 0.0046), cardiac involvement (83.33% vs. 22.22%, *p* = 0.0008), subcutaneous calcinosis (41.66 vs. 11.11., *p* = 0.01146), and claw hand deformity (25% vs. 11.11%, *p* = 0.00016) were significantly associated with the presence of PAH in our overlap cohort.

## 4. Discussion

In the present study, the clinical, laboratory and genetic features of SSc-myositis overlap syndrome were assessed in a Hungarian cohort of 39 patients. We can summarize our recent work as follows: (1) we confirmed that the genetic characteristics, namely, the HLA-DRB1 and DQA1 genotypes of the overlap patients, are clearly different from healthy individuals, but not significantly different from genetics of patients with anti-Jo-1 ASSD; (2) myositis-SSc overlap patients have a distinct and unique clinical phenotype, which seems similar in many ways with the phenotype of anti-Jo-1 ASSD patients. Besides the sclerodactyly, the main differences are the more frequent presence of dysphagia, Raynaud's phenomenon, and less frequent presence of livedo reticularis. (3) Distinct clinical parameters measured at disease onset (fever at diagnosis, subcutaneous calcinosis, cardiac involvement and claw hand deformity) were associated with the presence of PAH in the overlap patients.

To date there are not many studies reported in the literature of scleroderma-myositis overlap. Most of them are limited to a small number of overlap cases [4, 25–28]. The comparison of our data with the results of other workgroups is challenging, since despite the definitions of overlap syndromes being obvious, sometimes it is not clear, whether myositis is represented as muscle involvement of SSc or as a different disease. In view of the clinical picture, we found that the ratios of lcSSc and dcSSc were quite similar, whereas PM over DM dominance was detected regarding the IIM phenotype in the overlap patients. Besides sharing clinical features of both diseases, these patients may have characteristic autoantibody positivity. In the context of SSc-myositis overlap, autoantibodies against Pm-Scl protein complex are the most relevant [29]. In our Hungarian population, none of the autoantibodies could be identified as a dominant marker for this overlap syndrome. Interestingly, 12 cases of anti-DNA positivity were detected, but only 3 cases of lupus could be diagnosed. Other overlapping systemic autoimmune disorders, such as rheumatoid arthritis or Sjögren's syndrome, were not detected. There was no anti-Ku or anti-U1-RNP-positive patient in this cohort, which is a fact of interest because these are myositis-associated antibodies which can be found in overlap cases.

Anti-Jo-1-positive ASSD is a complex disease without well-established classification criteria, and the clinical picture of these patients could be similar to IIM-SSc overlap patients. Presumably, when myositis-specific antibodies (MSA) testing is not available, the majority of patients can be categorized into both disease groups. With our recent work, we could confirm that based on the comparable presence of HLA − DRB1∗03 and HLA − DQA1∗05 : 01 alleles, the two entities share similar genetic features too, which is evidently different from genetic results of healthy individuals. However, it should be emphasized that despite the unique anti-Jo-1 autoantibody positivity of our ASSD cohort, both ASSD and overlap diseases show intense heterogeneity regarding the organ involvement and the clinical picture. Nevertheless, it seems that dysphagia and RP are more characteristic, but livedo reticularis is less typical to IIM-SSc overlap than to anti-Jo-1-positive ASSD patients. The similarities of the two cohorts could be, at least partly, explained by the fact that all of our anti-Jo-1-positive patients had myositis, which could be different in other ASSD cohorts [30], since some groups are selecting ASSD patients based on the positivity of anti-Jo-1 test and the presence of at least one clinical finding between arthritis, myositis, and ILD. Therefore, development of new classification criteria of ASSD based on differential weights for various clinical, pathological, and serological variables might help to select appropriate patients to this disease and compare more unified cohorts.

The most frequently reported genetic associations with SSc are HLA − DRB1∗11, HLA − DQB1∗03 among European and North American Caucasians [16, 31, 32], and HLA DRB1∗03 : 01 and DQA1∗05 : 01 in myositis [14, 15]. It is also reported that distinct HLA associations have been described in IIM in different populations, clinical subgroups, and with specific clinical features; however, the strongest HLA associations are found when stratifying by autoantibody status [33]. In the setting of Caucasian myositis patients, anti-PM/Scl autoantibody was strongly associated with HLA − DQB1∗02 : 01, anti-Jo-1 with HLA − B∗08 : 01, and HLA − DRB1∗03 : 01 alleles [14]. Our overlap population did not have a unique autoantibody profile, and the presence of autoantibodies showed high variance (Figure 1). However, the genetic results showed quite similar data than those found in patients with anti-Jo-1 ASSD, which indicates a different link between genetic susceptibility, autoantibodies, and disease phenotype.

PAH is a significant factor of morbidity and mortality of patients with IIM and/or SSc. The occurrence ratio of PAH was 30.77% in our SSc-myositis cohort, which seems higher than those found in patients with SSc (7-12%) [34–36] and in myositis (5-17) [37–39]. This interesting finding could demonstrate that the presence of myositis might be associated with cardiac involvement in SSc [40–42]. PAH is often associated with extensive ILD as a secondary feature and does not differ clinically from idiopathic PAH. However, in our cohort, the presence of ILD was similar in the PAH-positive and PAH-negative group, supporting an alternative development of the pulmonary hypertension, but it should be noted that the extension and severity of ILD and fibrosis was not compared between the two groups in this study. Nevertheless, the results that the presence of fever at diagnosis, subcutaneous calcinosis, and claw hand deformity was associated with PAH in our cohort might facilitate more frequent assessments of PAH diagnostic procedures in selected cases, which could lead to early diagnosis and adequate treatment. We have found that the mortality results of the two groups did not show any significant associations, which could be accountable for the low number of patients enrolled and the short follow-up.

The possible limitations of this study should be acknowledged. This work was a single center study from a national myositis unit in Hungary, and the number of participants in the study was relatively low. The lower number of patients with genetic examinations could be a cause for selection bias, and determination of PAH was performed via noninvasive methods, which has lower specificity than Swan-Ganz catheterization procedure.

## 5. Conclusions

It can be concluded that SSc-myositis overlap syndrome forms a unique subgroup within the IIMs. The presence of lcSSc and dcSSc were comparable, whereas PM was the dominant type of myositis in the patients. The occurrence of PAH seems higher than in other subgroups and is associated with certain clinical features, such as fever at diagnosis, claw hand deformity, and subcutaneous calcinosis, drawing attention to more frequent diagnostic screening. SSc-IIM overlap patients show notable similarities with anti-Jo-1-positive ASSD patients regarding HLA alleles and major clinical characteristics with certain distinct exceptions. Further evaluation of larger multicentric cohorts with outcome results are required to assess more detailed features of SSc-IIM overlap disease.

## Figures and Tables

**Table 1:** Demographic, laboratory, and clinical results of SSc-myositis overlap vs. anti-Jo-1-positive patients.

	SSc-myositis overlap patients	Anti-Jo-1-positive patients [22]	*p* value
*Demographic data*			
Number of patients:	39	49	—
Male/female:	9/30	7/42	—
Average age at disease onset ± SD (youngest-oldest):	42 ± 14 (13-76)	43.4 ± 13.28 (18-70)	ns
Number/% of lcSSc	22/56.41	—	—
Number/% of dcSSc	17/43.59	—	—
Number/% of DM	3/7.69	—	—
Number/% of PM	36/92.31	—	—
Occurred simultaneously/initially diagnosed with myositis/scleroderma:	23/4/12	—	—
*Clinical results*			
ILD/alveolitis (no. of patients/%):	24/71.79	36/73.47	ns
Arthritis/arthralgia (no. of patients/%):	32/82.05	43/87.76	ns
Dysphagia (no. of patients/%):	26/66.67	6/12.24	<0.0001
Intestinal involvement except dysphagia (no of patients/%)	11/28.21	5/10.2	0.03
Fever (no. of patients/%):	7/17.95	21/42.86	ns
Raynaud's phenomenon (no. of patients/%):	38/97.44	32/65.31	<0.001
Renal involvement (no. of patients/%):	5/12.82	—	—
Cardiac involvement (no. of patients/%):	16/41.03	—	—
PAH (no. of patients/%):	12/30.77	—	—
Mechanic's hand (no. of patients/%)	5/12.82	16/32.65	ns
Subcutaneous calcinosis (no. of patients/%)	8/20.51	3/6.12	ns
Livedo reticularis (no. of patients/%)	2/5.13	16/32.65	<0.01
*Laboratory results*			
Average CK at diagnosis (U/l) ± SD	1542.37 ± 1975.33	3003.25 ± 3101.8	ns
Average LDH at diagnosis (U/l) ± SD	725.67 ± 360.58	922.33 ± 635.32	ns
SSA positivity (no. of patients/%)	9/7.69	17/34.69	ns
Average ESR at diagnosis (mm/h) ± SD	40.26 ± 23.72	24.24 ± 15.96	<0.01

SD: standard deviation; CK: creatine kinase; LDH: lactate dehydrogenase; SSA: Sjögren's-syndrome-related antigen A; ILD: interstitial lung disease; PAH: pulmonary arterial hypertension. Only age at diagnosis of myositis has Gaussian distribution in both groups; Welch's *d*-test was applied. Significance was calculated with independent samples *t*-test or Mann–Whitney test, according to the distribution. Normality of the distributions was checked using Shapiro-Wilk test. Symptoms and laboratory parameters were compared between the two groups with Fisher's exact test, applying Hochberg's correction for multiple comparisons.

**Table 2:** Comparison of patients with and without PAH.

SSc-myositis overlap	Patients with PAH		Patients without PAH		*p* value
*Demographic data*					
Number of patients	12		27		—
Male/female	2/10		7/20		ns
Average age at SSc diagnosis (years) ± SD	44.5 ± 15		41.1 ± 14.3		ns
Average age at myositis diagnosis (years) ± SD	44.5 ± 15.4		41.7 ± 14.5		ns
dcSSc/lcSSc	4/8		13/14		ns
Number of death (no. of patients/%)	4/10	40	2/17	11.76	ns
*Clinical data*					
ILD (no. of patients/%)	9	75	21	77.78	ns
Arthritis (no. of patients/%)	10	83.33	22	81.48	ns
Raynaud's phenomenon (no. of patients/%)	12	100	26	96.3	ns
Dysphagia (no. of patients/%)	9	75	17	62.96	ns
Fever (no. of patients/%)	5	41.67	2	7.41	0.0046
Cardiac involvement (no. of patients/%)	10	83.33	6	22.22	0.0008
Calcinosis (no. of patients/%)	5	41.66	3	11.11	0.01146
Claw hand deformity (no. of patients/%)	3	25	3	11.11	0.00016

SD: standard deviation; ILD: interstitial lung disease; PAH: pulmonary arterial hypertension; ns: not significant; stepwise discriminant analysis with Wilks method was performed. Continuous and binary variables were investigated. Significances of the association between PAH and the outcome of the disease were calculated with Fisher's exact test.

## Data Availability

All patients are followed by the Department of Clinical Immunology and Rheumatology at the University of Debrecen, Hungary. Medical files of the patients (available only for our myositis workgroup because of ethical reason) were reviewed by the help of MedSol Database. Studies are available upon request from the corresponding author. The data are not publicly available.
